# System-Level Biochip for Impedance Sensing and Programmable Manipulation of Bladder Cancer Cells

**DOI:** 10.3390/s111111021

**Published:** 2011-11-23

**Authors:** Cheng-Hsin Chuang, Yao-Wei Huang, Yao-Tung Wu

**Affiliations:** Department of Mechanical Engineering, Southern Taiwan University, Tainan 71005, Taiwan; E-Mails: z5972324@hotmail.com (Y.-W.H.); sun10192004@yahoo.com.tw (Y.-T.W.)

**Keywords:** dielectrophoresis (DEP), single cell, manipulations, microstructure

## Abstract

This paper develops a dielectrophoretic (DEP) chip with multi-layer electrodes and a micro-cavity array for programmable manipulations of cells and impedance measurement. The DEP chip consists of an ITO top electrode, flow chamber, middle electrode on an SU-8 surface, micro-cavity arrays of SU-8 and distributed electrodes at the bottom of the micro-cavity. Impedance sensing of single cells could be performed as follows: firstly, cells were trapped in a micro-cavity array by negative DEP force provided by top and middle electrodes; then, the impedance measurement for discrimination of different stage of bladder cancer cells was accomplished by the middle and bottom electrodes. After impedance sensing, the individual releasing of trapped cells was achieved by negative DEP force using the top and bottom electrodes in order to collect the identified cells once more. Both cell manipulations and impedance measurement had been integrated within a system controlled by a PC-based LabVIEW program. In the experiments, two different stages of bladder cancer cell lines (grade III: T24 and grade II: TSGH8301) were utilized for the demonstration of programmable manipulation and impedance sensing; as the results show, the lower-grade bladder cancer cells (TSGH8301) possess higher impedance than the higher-grade ones (T24). In general, the multi-step manipulations of cells can be easily programmed by controlling the electrical signal in our design, which provides an excellent platform technology for lab-on-a-chip (LOC) or a micro-total-analysis-system (Micro TAS).

## 1. Introduction

Bladder cancer is reported as the fourth most common type of cancer in men and the eighth most common one in women. During the diagnosis of bladder cancer, the identification of the grade needs to be taken into account when deciding the treatment. Cystoscopy is the surest way to examine the grade of bladder cancer from a biopsy of the lining of the bladder; however, the patient may need anesthesia for this procedure. In addition to cystoscopy, a few biomarkers have been developed for urine tests, yet their sensitivity and selectivity remain unsatisfactory. Therefore, a high accuracy, non-invasive and *in vitro* method for the determination of the stage of bladder cancer is necessary to help bladder cancer patients. Recently, there has been considerable and growing interest in an electrical detection method and dielectrophoretic (DEP) manipulations for cell-based biochips because both are intrinsically electrical and microfluidic compatible. In 1999, Milner *et al.* [1] proposed an impedance technique for detecting a dielectrophoretic collection of microbiological particles by two coplanar microelectrodes. They found that the impedance depended on the number of particles captured by DEP force. Similarly, a chip with an interdigitated array microelectrode (IDAM) was most frequently utilized for dielectrophoretic impedance measurement (DEPIM) methods, such as for determination of the viability of *E. coli* by Suehiro *et al.* in 2003 [2], the death of yeast cells by Markx *et al.* in 2008 [3] and separation and detection of different-size microparticles by Ahn *et al.* in 2008 [4]. The DEPIM method can capture bioparticles either at the edge of the microelectrodes or in the gap between a pair of interdigitated electrodes by positive and negative DEP force, respectively, and simultaneously measure the impedance of captured bioparticles. Although DEPIM represents a fast and sensitive way for cell-based detection compared with conventional fluorescent detection, the impedance signal usually depends on the number of cells captured by DEP force, which is difficult to control with the IDAM design. In addition, the AC signal for DEP manipulations needs to be applied to the IDAM as an impedance measurement; according to the results, DEPIM is not suitable for long-time monitoring of living cells due to the effects of joule heating and the high intensity electric field which could cause cell damage in a short time. In 2007, Zhang *et al.* [5] proposed a different design for impedance measurement with single-cell resolution; he separated the electrodes for individual purposes of DEP trapping and impedance measurement; thus, long-term monitoring of dynamic process of endothelin-1-induced cardiomyocyte hypertrophy could be achieved. In the meanwhile, Chuang [6,7] demonstrated a DEP chip with multilayer electrodes and a micro-cavity array for trapping single cells in a micro-cavity by negative DEP force and sequentially sensing their impedance. In Chuang’s work, the impedance measurement of trapped cells in a micro-cavity did not have to apply DEP force due to the enhancement of the positioning and immobilization by the microstructure effects. Recently, Yun [8] and Jang [9] also continued using separation of DEP manipulations electrodes and impedance sensing electrodes for breast cancer cells (MCF7) and HeLa cells. Although the concept of single-cell impedance sensing has been demonstrated by several researchers, only a single cell can be measured in a time experiment on a chip that makes the advantage of rapidly sensing without meaning due to the fact the number of examined cells is small. In this study, we propose a system-level biochip for cell manipulations and impedance sensing in a 3 × 3 array by PC-based programming control and data acquisition. This biochip can trap cells in a micro-cavity array with single-cell resolution for impedance sensing, and then individually release the identified cells back to the flow chamber for recollection. As mentioned before, the most specific (90%) diagnostic test for bladder cancer is invasive (cystoscopy) in conjunction with cytology, however, the sensitivity is rather low (40–60%) particularly in the detection of low-grade, low stage bladder cancer cells. In order to detect the low-grade bladder cancer cells, two different-grade bladder cancer cell lines, (TSGH8301, grade II and T24, grade III) were utilized to detect them by an impedance measurement method. The second grade bladder cancer cells look like normal cells therefore missjudgments can frequently happen in cytology. Consequently, we tried to provide a more rapidly and noninvasive way for differentiation of different-grade bladder cancer cells.

## 2. Theory and Simulation

### 2.1. Theory of Dielectrophoresis and Impedance Measurements

The time-averaged dielectrophoretic force acting on a spherical particle was immersed in a medium and exposed to a spatially non-uniform electric field [10]. The dipole component of the DEP force is expressed as:

(1) $$F_{\mathrm{DEP}}=2{\pi ɛ}_{m}R_{p}^{3}\;\text{Re}\left[K(\omega )\right]\nabla \;E_{\mathrm{rms}}^{2}$$

where *ɛ_m_* is the electrical permittivity of the surrounding medium, *R_p_* is the radius of the particle, $\left|{\Delta E}_{\mathrm{rms}}^{2}\right|=\sqrt{{\Delta E}_{x}^{2}+{\Delta E}_{y}^{2}+{\Delta E}_{z}^{2}}$ is the gradient of the square of applied electric field magnitude, and *K* (*ω*) is the frequency dependent Claussius-Mosotti (CM) factor for a dielectric uniform sphere, such as a bead; it is expressed as:

(2) $$K(\omega )=\frac{{ɛ}_{p}^{*}-{ɛ}_{m}^{*}}{{ɛ}_{p}^{*}+{2ɛ}_{m}^{*}}$$

where ɛ* is the complex permittivity of the medium (m) or particle (p) and defined by:

(3) $${ɛ}^{*}=ɛ-j\frac{\sigma }{\omega }$$

where ɛ is the permittivity of the medium or particle, σ is the conductivity of the medium or particle, and j is $\sqrt{1}$. Hence, the CM factor can be viewed as the ratio of electrical conductivities between the particle and the medium at a low frequency; on the other hand, it can be regarded as the ratio of permittivities between the particle and the medium at high frequency. The sign of the CM factor shows whether it is positive-DEP or negative-DEP. When the real part of the CM factor is a positive value, $\text{Re}\left[\frac{{ɛ}_{p}^{*}-{ɛ}_{m}^{*}}{{ɛ}_{p}^{*}+{2ɛ}_{m}^{*}}\right]>0$, particles suspended in the medium will be moved toward the region possessing a high intensity electric field by the positive-DEP force. Conversely, when $\text{Re}\left[\frac{{ɛ}_{p}^{*}-{ɛ}_{m}^{*}}{{ɛ}_{p}^{*}+{2ɛ}_{m}^{*}}\right]<0$, the DEP force will move particles toward the region possessing a low intensity electric field, the so-called negative DEP. Furthermore, as $\text{Re}\left[\frac{{ɛ}_{p}^{*}-{ɛ}_{m}^{*}}{{ɛ}_{p}^{*}+{2ɛ}_{m}^{*}}\right]=0$, the DEP force will be equal to zero, which means the suspended particles will not be affected by the DEP force (the corresponding frequency of the AC signal is called the cross-over frequency). Thus, in a non-uniform electric field, whether a positive or negative DEP force acts on the particles depends on the sign of the CM factor. Furthermore, the magnitude of the DEP force is determined by the imposed gradient of the square of applied electric field magnitude at the particle position, as well as by the radius of the particle. Usually, DEP force is only valid for a certain range, due to the fact that ∇E^2^ decreases rapidly away from electrode. Therefore, the gradient of the square of the applied electric field, ∇E^2^, will be evaluated by a simulation method in the next section.

Regarding of impedance measurement, impedance (Z) is an important parameter of electronic components defined as the total opposition of device or component offers to the flow of an alternating current (AC) at a given frequency. For the total impedance of an undetermined object, the total current is usually measureable by a specific instrument, therefore, the total impedance could be further calculated by *Z* = *V* / *I*, where *V* is the applied voltage and *I* is the total current. In addition, the total current could be calculated by integration of current density, *J*, as *I* = ∫*_s_ JdS*, where current density *J* is defined as the distribution of flow of charge and *S* is defined as a surface area of the current passing through. As a result, the total current density could be regarded as being inversely proportional to impedance if the applied voltage and surface area are constant. In the simulation section, the current density for the impedance measurement of cells will be investigated.

### 2.2. Simulation of Nonuniform Electric Field

**Figure 1.**
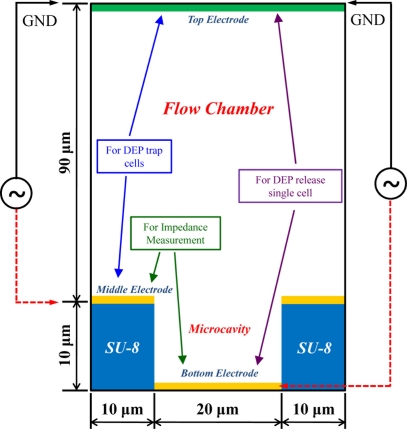
The 2D and 3D model of multilayer electrodes DEP chip; the height of the flow chamber is 90 μm, the thickness of SU-8 layer is 10 μm and the diameter of the cavity is 20 μm.

The 3D model of the present multi-electrode DEP chip was simulated by the CFD-ACE+ software (ESI Group, France) and the parameters for medium, SU-8 and cell are listed in Table 1. The structure of the DEP chip consisted of an ITO top electrode, flow-chamber, middle electrode on the SU-8 surface and the 3 × 3 bottom electrode array under the SU-8 micro-cavities. There were three layers of metal electrodes for three individual purposes; one is to trap cells in the micro-cavity array by negative DEP force generated by the top and middle electrodes, another is impedance measurement by the middle and bottom electrodes separated by the SU-8 layer, and the final one is to release single cells by negative DEP force generated by top and bottom electrodes, as shown in the Figure 1.

**Figure 2.**
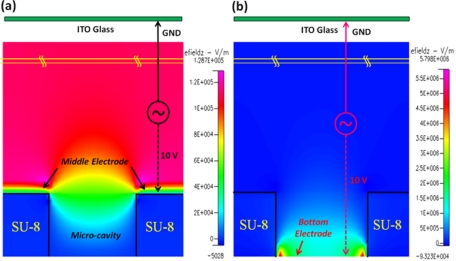
**(a)** The contour of electric field as applied the AC signal to the middle and top electrodes for trapping cells; **(b)** The contour of electric field as applied the AC signal to the bottom and top electrodes for releasing cells.

**Figure 3.**
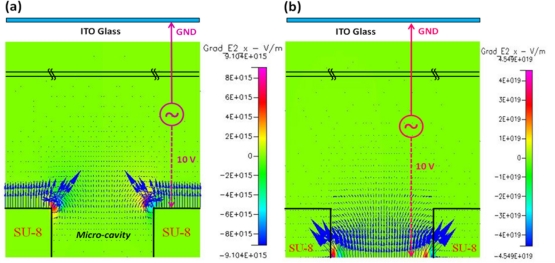
The vertical component of ∇E^2^: **(a)** Trapping cells by applied AC signal to top and middle electrodes; **(b)** Releasing cells by applied AC signal to top and bottom electrodes.

As the simulation results show, the highest electric field was near the top of the middle electrode surface, conversely; a weak electric field occurred in the SU-8 micro-cavity. Hence, cells would be moved into the micro-cavity by negative DEP force for further cell analysis, as shown in Figure 2(a). According to our experiments; the cells trapped in the micro-cavity could maintain viability and stability for at least 3 h in the micro-cavity without applied DEP voltage. When the cells were trapped in the micro-cavities upon the bottom electrodes, we could apply the AC signal to the top and bottom electrodes to individually release trapped single cells by negative DEP force. In Figure 2(b), the contour of electric field, E, shows that the highest electric field density occurred near the edge of bottom electrode and the lowest one took place in the flow chamber; thus, programmable control of which trapped cell should be released can be done by switching the AC signal to the corresponding bottom electrode. As mentioned in the DEP theory, the DEP force directly depends on the gradient of the square of electric field, ∇E^2^, Figure 3(a,b) show the contour of vertical component of ∇E^2^ for trapping and releasing cells, respectively. Note that the arrows indicated in Figure 3 represent the magnitudes and vectors pointing in the direction of the steepest grade of E^2^ at that point. Consequently, the largest DEP force happened at the edge of middle electrode as trapping cells and the edge of bottom electrode as releasing cells.

### 2.3. Simulation of Current Density

The impedance sensing was conducted by applying a lower voltage (1V) to the middle electrode upon the SU-8 surface and grounding the bottom electrode under the SU-8 micro-cavity. The electric field under impedance sensing therefore differs from one of conventional planar counter electrodes. In order to investigate the impedance sensing within the micro-cavity structure, the same 2D model as indicated in Figure 1 was analyzed by the COMSOL software. Because the two impedance electrodes (the middle and bottom electrodes) were separated by an SU-8 insulator layer, the current could only pass through the medium between the two impedance electrodes. In our simulations, the total current density of the flow chamber area could be directly calculated by the simulation tool; hence, we took the total current density as the inverse index of the resulting impedance.

**Figure 4.**
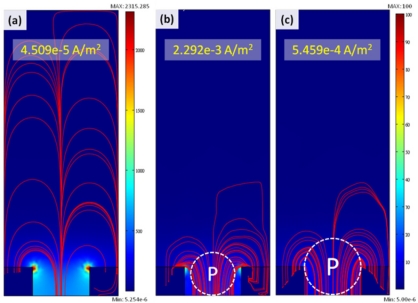
The distribution of current density as impedance measurement by applying voltage to middle and bottom electrodes; **(a)** without particle in the SU-8 cavity; **(b)** and **(c)** with 15 μm and 20 μm cell in the SU-8 cavity, respectively.

In order to investigate the effects of cell size and its dielectric properties on the current density, we established three typical models, as illustrated in Figure 4: one is only medium without cell in the micro-cavity, the other two are single cells immobilized in the micro-cavities, but the cell sizes refer to the real cell sizes of T24 and TSGH8301 (15 μm and 20 μm, respectively). As far as we know, the dielectric properties of bladder cancer cells (T24 and TSGH8301) have not be explained in past studies; therefore, we utilized the HeLa cell [11] as the reference of cell dielectric properties, as indicated in Table 1. Due to the fact a cell consists of a cell membrane and cytoplasm, the modeling of the cell was simulated as a layered spherical particle. The thickness of the thin outer layer of the cell membrane, 50 nm, was limited by the element number as the finite element analysis operated in a personal computer (PC) though the thickness of the cell membrane is usually less than 5 nm; a similar issue was also mentioned by Malleo *et al.* [12]. From Figure 4, the distribution of current density, from applying an AC signal of 1V at 1 kHz, exhibits an obvious difference with and without the cell in the micro-cavity. In addition, the total current density for the case without a cell in the micro-cavity is lower than the other two cases with a cell in the micro-cavity. Furthermore, a higher current density can be calculated for the micro-cavity possessing a cell with smaller size. Consequently, the impedance values from large to small can be interpreted as Z_without-cell_ > Z_20μ__m-cell_ > Z_15μ__m-cell_. This result will be verified by experimental measurement.

**Figure 5.**
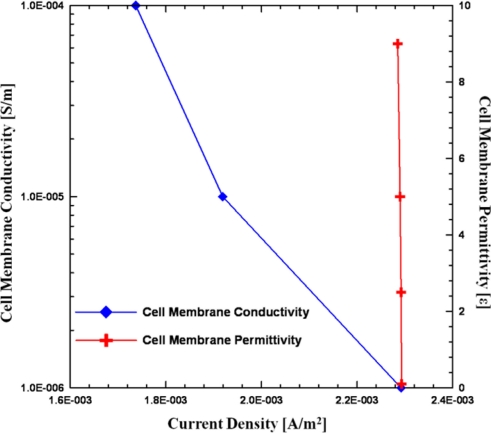
The variation of total current density as changing the cell membrane conductivity and permittivity.

Besides size effects, other important factors of impedance measurement are the dielectric properties of the cells. In order to evaluate the influence of the conductivity and permittivity of the cell membrane and cytoplasm, a series of parametric studies was performed, as listed in Table 2 and plotted in Figure 5. From the parametric simulation results, by increasing the conductivity of the cell membrane, the total current density is decreased, although its variation is smaller than that of the size effect. On the other hand, the permittivity of cell membrane seems independent with total current density as the vales varied from 0.1 to 9 [12]. Therefore, the impedance sensing of cells depends on its size and the conductivity of cell membrane, however, the influence of cell size is more significant than cell membrane conductivity.

## 3. Fabrication of DEP Chip

**Figure 6.**
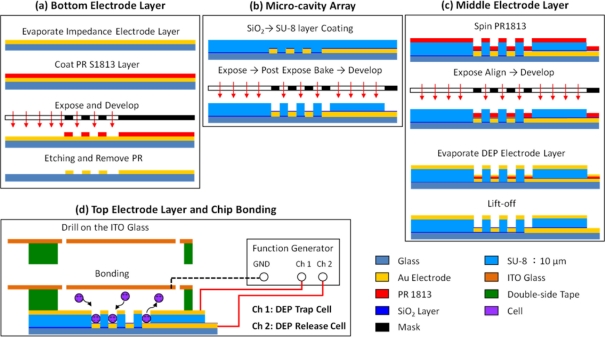
The microfabrication processes of DEP chip.

**Figure 7.**
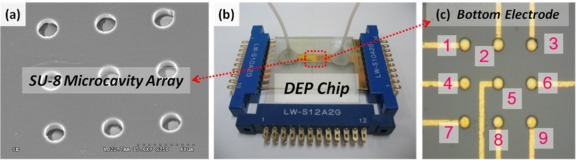
**(a)** the SEM image of the 3 × 3 micro-cavity array; **(b)** the photograph of the entire DEP chip; **(c)** optical image of bottom electrodes (resealing electrode) under micro-cavity array.

The DEP chip with multilayer electrodes consisted of three parts, as shown in Figure 6. The bottom electrodes for individually releasing cells were patterned first on a 40 × 70 mm^2^ microscope slide, as shown in Figure 6(a). The glass slide was cleaned in acetone, followed by the use of a methanol solution, then an ultrasonic cleaning machine for 5 min, and finally dried with a N_2_ gun and dried for an additional 30 min at 225 °C. The layout of 3 × 3 individual bottom electrodes is shown in Figure 7(c); the diameter of each bottom electrode was 20 μm. The middle part consisted of a cavity-type 3D microstructures array and the middle electrode for DEP trapping; these comprised a thick photoresist layer, SU-8 and a metal layer, Au, respectively. The 3 × 3 micro-cavity array was first patterned on the SU-8 layer, as shown in Figures 6(b) and 7(a). The diameter, spacing and depth of the micro-cavity array were designed as 20 μm, 20 μm and 10 μm, respectively. Before depositing the metal layer onto the SU-8 surface, the same photo-mask for the micro-cavity was used again for patterning the positive photo-resist S1813 in the micro-cavity for the later lift-off process. Then, 300 Å of chromium and 700 Å of gold were evaporated sequentially onto the SU-8 microstructure by E-beam evaporator under temperature control for the avoidance of SU-8 reflow; finally, the metal layer at the bottom of the micro-cavity was lifted off by immersion in acetone, as shown in Figure 6(c). The last part was a rectangular flow chamber, with the dimensions of W × L × H = 7 mm × 50 mm × 100 μm, formed by double-sided tape attached to the ITO glass. The advantages of using double-sided tape were its ease of patterning and the good quality of bonding between the upper ITO glass and the SU-8 layer, as shown in Figure 6(d). The finished multilayer electrodes DEP chip is shown in Figure 7(b).

## 4. Experimental Setup

**Figure 8.**
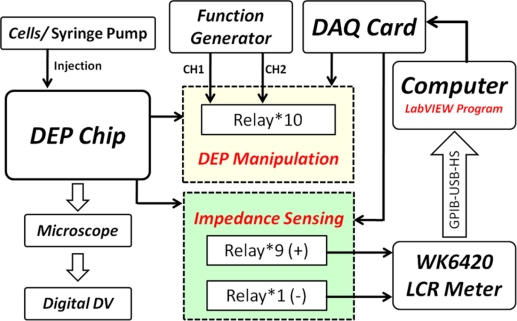
The experimental setup for DEP trapping, releasing and impendence sensing of cells.

The experimental setup is shown in Figure 8. We utilized a syringe pump (KDS-210, KD Scientific) to control the flow rate. The AC signal was generated by a function generator (AFG3022, Tektronix) for DEP trapping and releasing. A digital DV (HDR-XR350, Sony) was mounted on a biological microscope (BX51, Olympus) for monitoring the DEP force acting on the cells and capturing the in-situ image for post image processing. The impedance amplitude was recorded for frequencies from 1 kHz to 100 kHz with continuously scanned spectra using the LCR meter (WK6420A, Wayne Kerr). In order to demonstrate the manipulation of the cells, two cell suspensions of human bladder cancer cell lines (T24 and TSGH8301) were utilized and immersed in a sucrose solution (8.63% in weight percentage) in this study. In addition, the conductivity of cell suspension was controlled in a proper range for DEP manipulation; we utilized a conductivity meter (SC-170, Suntex) to measure the cell suspensions. Furthermore, the isotonic sucrose solution can maintain the cells’ viability for at least 4 h.

**Figure 9.**
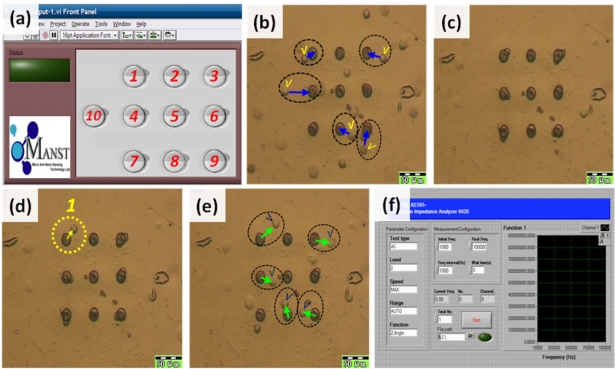
Optical micrographs demonstrating the trapping and programmable releasing of single cells: **(a)** LabVIEW program for manipulation of cells, **(b)** cells suspended in the micro-cavity array, **(c)** trapping cells in the micro-cavity array by switch on No. 10 with an AC signal in the negative-DEP range, **(d)** show the target (arrow) cell released by switch on No.1 button, **(e)** all other trapped cells were released by switch on No. 10 button with an AC signal in the positive-DEP frequency range, **(f)** LabVIEW program for impedance sensing of cells.

In this work, all of the procedures for DEP manipulation were performed by a PC-based LabVIEW programming system. The DEP chip includes nine independent bottom electrodes and one ITO top electrode, connected with the circuit board and the DAQ system for trapping and programmably releasing single cells. The DAQ system provides for the programmable release of single cells by the LabVIEW software program. The LabVIEW program can individually control ten switches for introducing the AC signals into 10 electrodes, including a middle electrode for trapping cells (No. 10), and nine individual bottom electrodes under the micro-cavities for releasing trapped cells (No. 1–9) as indicated in Figures 7(c) and 9(a). Another LabVIEW program for controlling the impedance sensing is shown in Figure 9(f); therefore, the impedance measurement for each micro-cavity in the DEP chip can be automatically recoded.

## 5. Results and Discussion

### 5.1. Cell Manipulation

The frequency ranges of positive and negative DEP for cells suspended in a sucrose solution (8.63% weight percent crystalline sucrose in 2DI water) after a series of DEP experiments, are listed in Table 3. The negative DEP force occurred at a low frequency range and the positive DEP force was generated at a high frequency range. In addition, the cross-over frequency was measured by the observation on the pulled-out phenomena for a trapped cell. Firstly, by applying an AC signal under 10 V_PP_ and 50 KHz cells were trapped into the microcavities, then, tuned the frequency up gradually until the trapped cells began to move up. We took the corresponding frequency as the lower bound of cross-over frequency. Usually, the trapped cells could be seen moving up and down for a certain frequency range as the frequency kept increasing. Finally, the cells moved to the middle electrode surface upon SU-8, the corresponding frequency was denoted as the upper bound of cross-over frequency. Thus, the upper and lower bound of cross-over frequency can be identified based on the DEP chip with microstructure array.

The cell manipulations were demonstrated by sequential operation, as shown in Figure 9. First, the cells were injected into the flow chamber by syringe pump at a rate of 0.5 μL/min for about 1 min to fill the flow chamber, as indicated in Figure 9(b). Then, the AC voltage with 10 V_PP_ at 20 kHz was applied to the top and middle electrodes. The cells moved into the micro-cavities by negative DEP force, as shown in Figure 9(c). Finally the target cells could be individually released from the micro-cavity by switching the AC voltage with 10 V_PP_ at 80 kHz to the top and bottom distributed electrodes. After the recollection of target cells, other cells could be easily popped out by applying the AC voltage with 10 V_PP_ at 200 kHz to the top and middle electrode by positive DEP force and flushed away for the next run, as shown in Figures 9(d,e). Consequently, this DEP chip provided a platform for the manipulation of cells and cell-examination purposes.

### 5.2. Impedance Sensing

**Figure 10.**
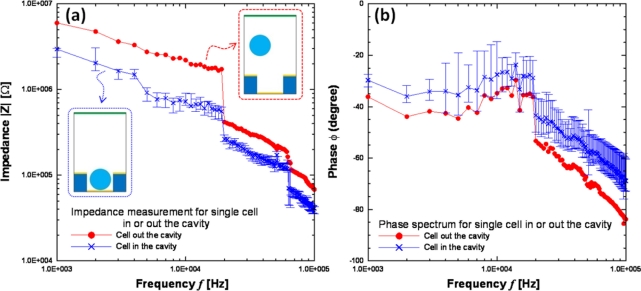
Impedance measurement results for with or without TSGH8301 cell in the micro-cavity array; the applied voltage was 1 V and the frequency range was swept from 1 K to 100 KHz.

**Figure 11.**
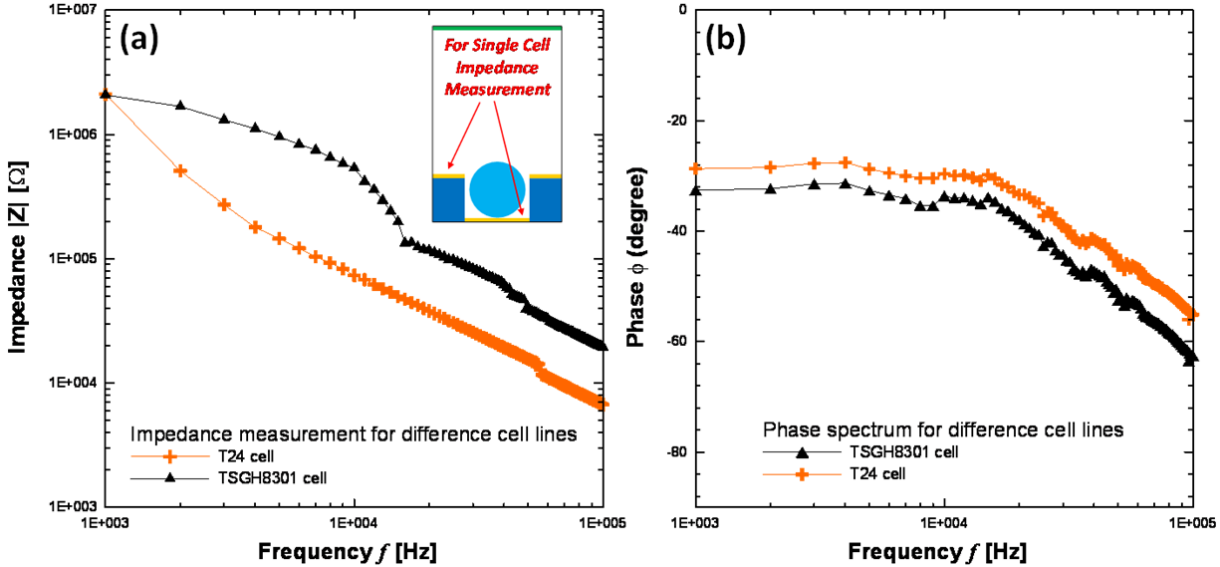
Impedance measurement results for difference bladder cancer cells (T24 & TSGH8301); the applied voltage was 1 V and the frequency range was swept from 1 K to 100 KHz.

The impedance measurement can be carried out by the middle and bottom electrodes after cells have been trapped in the micro-cavity. The cells were immersed in a sucrose solution and pumped into the flow chamber by a syringe pump. After these suspended cells flowed over the block upon the micro-cavities array in the flow chamber, the AC signal was applied for DEP trapping. When the cells were trapped in the micro-cavities upon the impedance electrodes, we flushed the suspended cells away from the cavity array to prevent parasitical effects during impedance measurement. Before the impedance measurement by LabVIEW program integrated with LCR meter, the syringe pump and the AC signal were stopped. Two different-grade bladder cancer cell lines (grade III:T24 and grade II:TSGH8301) were utilized for the impedance sensing. As the experimental results in Figure 10 show, the impedance decreased when cells were trapped in the micro-cavity array, which is consistent with simulation results. The impedance measurement results for different-grade bladder cancer cell lines are shown in Figure 11. The lower-grade bladder cancer cells (TSGH8301) possess higher impedance than the higher-grade ones (T24). Besides, the impedance magnitudes decreased as the frequency increased for all conditions, which indicated a capacitor characteristic of our DEP chip. Another impedance investigation on the human breast cancer cell lines [13] (MCF-7, MCF-MB-231, and MDA-MB-435) of different pathological grades also indicated similar tendency higher-grade breast cancer cell line possessed lower impedance magnitude. In addition, pathological change could induce a series of variations in membrane potential, ion channel as well as membrane protein, *etc.* Therefore, different-grade cancer cells could display different impedance. Although we know that grading in cancer is a measurement of the cell appearance in tumors and other neoplasms, but the impedance measurement method also has demonstrated differentiable variations between different-grade bladder cancer cell lines. Consequently, the impedance measurement method for the differentiation of different-grade bladder cancer cells is promising, but still needs further calibration on the effects of cell size and contact resistance of electrode by an equivalent circuit model. In this study, not only cell manipulations but also the impedance data acquisition in a micro-cavity array were performed by the PC-based programming so that the examination time for one run can be reduced to 3 min. Thus, if the DEP chip integrates with a circulating pumping system, the total number of examined cells could be greatly improved with multi-run tests.

## 6. Conclusions

We have designed and fabricated a DEP chip with multi-layer electrodes and micro-cavity array used for the trapping, programmable releasing and impedance measurement of cells at a single-cell level. All of the operations were integrated by a PC-based LabVIEW program; therefore, we have demonstrated a system-level biochip for cell analysis on a microchip. From the impedance measurement results, the impedance of low-grade bladder cancer cells is higher than the high-grade ones. Consequently, this microchip not only provides an efficient way to immobilize cells in the micro-cavity for a long period of time without applying DEP force, but also easily discriminates the different-grade bladder cell lines based on an on-chip impedance measurement, so that the cell identification and recollection can be achieved by this enabling technology.

## Figures and Tables

**Table 1.** Material Properties for Simulation.

				**Hela Cell [11]**	
**Properties/Materials**	**Medium**	**Au**	**SU-8**	**Cell Membrane**	**Cytoplasm**
Density ρ (kg/m^3^)	1,000	19,320	1,194	997	997
Viscosity η (kg/m·s)	8.92 × 10^−4^	-	-	-	-
Conductivity σ (S/m)	2 × 10^−4^	455 × 10^5^	2 × 10^−3^	10 × 10^−7^	0.435∼1.25
Relative Permittivity ɛ	80	69 × 10^−1^	37 × 10^−1^	2.5 (assumed as the same with latex bead)	35∼60

**Table 2.** Parametric studies for simulation of cell impedance.

**Cell Membrane**		**Cytoplasm**		**Current Density (A/m^2^)**

**Conductivity σ (S/m)**	**Relative Permittivity ɛ**	**Conductivity σ (S/m)**	**Relative Permittivity ɛ**	
			35	2.292 × 10^−3^
10 × 10^−7^	2.5	1	47	2.292 × 10^−3^
			60	2.292 × 10^−3^

		0.435		2.292 × 10^−3^
10 × 10^−7^	2.5	1	47	2.292 × 10^−3^
		1.25		2.292 × 10^−3^

	0.1			2.293 × 10^−3^
10 × 10^−7^	2.5	1	47	2.292 × 10^−3^
	9 [12]			2.285 × 10^−3^

10 × 10^−7^		1	47	2.292 × 10^−3^
10 × 10^−6^	2.5			1.919 × 10^−3^
				
10 × 10^−5^				1.737 × 10^−3^

**Table 3.** Frequency range of negative DEP, positive DEP and cross-over frequency for TSGH8301 (grade II) and T24 (grade III) cells suspended in the medium.

**Sample**	**Cell Size (Diameter/μm)**	**Negative DEP (kHz)**	**Cross-over Frequency (kHz)**	**Positive DEP (kHz)**
TSGH8301	20 ± 3	10∼50	140∼180	180∼1,000
T24	15 ± 3	10∼50	150∼200	200∼1,000
